# Sirtuin 1: A Dilemma in Transplantation

**DOI:** 10.1155/2020/9012980

**Published:** 2020-04-25

**Authors:** Sara Assadiasl, Nuala Mooney, Bahareh Mohebbi, Yousef Fatahi, Narjes Soleimanifar

**Affiliations:** ^1^Molecular Immunology Research Center, Tehran University of Medical Sciences, Tehran, Iran; ^2^Human Immunology and Immunopathology, Inserm UMR 976, Paris, France; ^3^Institut de Recherche Saint Louis, Sorbonne Paris Cité, Université Paris Diderot, Paris, France; ^4^Department of Pharmaceutical Nanotechnology, Faculty of Pharmacy, Tehran University of Medical Sciences, Tehran, Iran; ^5^Nanotechnology Research Center, Faculty of Pharmacy, Tehran University of Medical Sciences, Tehran, Iran

## Abstract

Sirtuin 1, a member of sirtuin family of histone deacetylase enzymes, has been implicated in a variety of physiologic and pathologic events, including energy metabolism, cell survival, and age-related alterations. In view of the anti-inflammatory properties of sirtuin 1 along with its protective role in ischemia reperfusion injury, it might be considered as contributing to the promotion of transplantation outcome. However, the potential ability of sirtuin 1 to induce malignancies raises some concerns about its overexpression in clinic. Moreover, despite the findings of sirtuin 1 implication in thymic tolerance induction and T regulatory (Treg) cells survival, there is also evidence for its involvement in Treg suppression and in T helper 17 cells differentiation. The identification of sirtuin 1 natural and synthetic activators leads to the proposal of sirtuin 1 as an eligible target for clinical interventions in transplantation. All positive and negative consequences of sirtuin 1 overactivation/overexpression in the allograft should therefore be studied thoroughly. Herein, we summarize previous findings concerning direct and indirect influences of sirtuin 1 manipulation on transplantation.

## 1. Introduction

Transplantation is considered as one of the most significant improvements in treating end-stage organ failure. Nowadays, solid organ transplantation, in particular, kidney and liver transplantations, is performed in many countries, and hematopoietic stem cell transplantation (HSCT) has been recognized as a therapeutic option for bone marrow-derived malignancies and insufficiencies [1]. The continuously growing number of transplantations is partly due to the increasing rate of certain chronic diseases (e.g., hypertension and diabetes) and the prolonged life expectancy of the human population; therefore, there is an urgent need to improve survival of donated organs, to promote recipients survival, and to improve the quality of life of transplant patients [2]. On the contrary, an extremely limited pool of matched donors and the transplantation of mismatched allografts (e.g., haploidentical and one-locus mismatch HSCT and various solid organ transplantations) make posttransplantation care more and more challenging.

The current strategy to improve graft survival requires life-long administration of immunosuppressive (IS) drugs in order to prevent graft loss due to alloreactivity. However, side effects of certain immunosuppressive treatments can lead to the metabolic disorders, an elevated infection rate, and an increased risk of malignancies [3]; hence, there is a need to develop novel therapies in order to prolong allograft survival. Recently, there have been some efforts to target certain molecules for specific inhibition of alloimmune responses [4]; in addition, various tolerance induction methods are studied in clinical trials [5]; a novel strategy may be envisaged by manipulating certain molecular pathways involved in tissue maintenance and hypoxia resistance [6]. For this purpose, sirtuin 1 (Sirt1) as a molecule with tissue protective potential might be a considerable candidate. Recently, there have been studies indicating advantages of Sirt1 expression in ischemia reperfusion injury models [7–9]; moreover, this molecule's anti-inflammatory effects have been described in various diseases [10–12]. In addition, there are some controversial findings implying the direct impact of Sirt1 on T cell subsets differentiation and function [13], while there is evidence of its involvement in certain malignancies [14].

Despite potential limitations, the availability of Sirt1 activator and inhibitor agents [15] makes it an appealing target to investigate in transplantation; therefore, we review the advantages and disadvantages of Sirt1 overexpression/overactivation in allograft tissue with the aim of providing an insight into its application (or not) as a supplementary substance to improve graft survival.

## 2. Sirtuin 1

**Figure 1:**
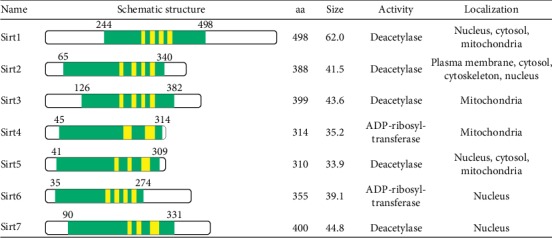
Schematic overview of human Sirt1–7 with a highly conserved catalytic core comprising NAD-dependent catalytic domain (NAD-binding pocket) (green) and zinc-binding domains (yellow). It also represents the amino acids (aa) number, molecular size (kDa), enzymatic activity, and intracellular localization of Sirt family.

Sirtuins (Silent Information Regulator Two proteINs) are nicotinamide adenine dinucleotide- (NAD-) dependent enzymes belonging to the class III of histone deacetylases. They are highly conserved molecules found in most species from unicellular organisms to eukaryotes. Sirt1 is the mammalian functional and structural homologue of the yeast Silencing Information Regulator (SIR2) which was first described in 1999-2000 as a life-prolonging factor [16]. Since then, more studies identified seven distinct proteins of the sirtuin family in human referring them as sirtuins 1–7 with Sirt1 being the most studied member of the family [17] (Figure 1). Sirtuins are present in almost all subcellular compartments. Sirt2 is found in the cytoplasm, while Sirt6 and Sirt7 have been traced in the nucleus and Sirt3, Sirt4, and Sirt5 are located in the mitochondria. Sirt1 is mainly concentrated in the nucleus although there are some reports about its occasional leakage into the cytoplasm [18, 19].

**Figure 2:**
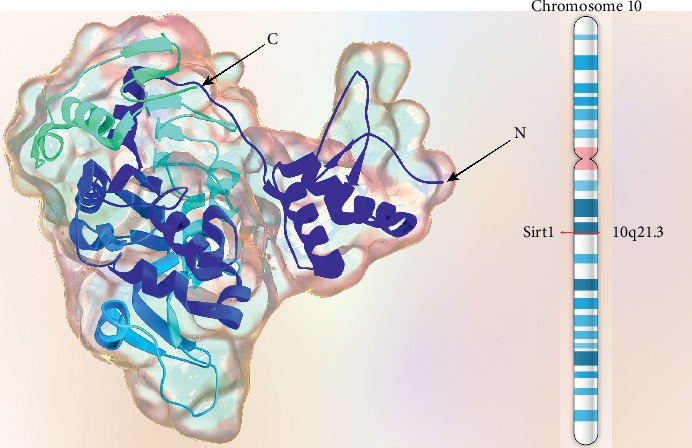
Schematic structure of SIRT1 molecule and its chromosomal location.

Human Sirt1 gene is located on chromosome 10q21.3 consisting of 8 introns and 11 exons. It is about 33 Kb long and has nontranslating regions in both 5′ and 3′ends. Sirt1 protein composes 747 amino acid residues. The structure includes a catalytic core region flanked by variable N- and C-terminals. The catalytic core is composed of approximately 254 amino acids including a homologous Rossmann-fold domain characteristics of NAD+/NADH binding proteins, a smaller zinc-binding domain (residues 362–419), cofactor binding loop, and several other loops connecting the two major domains [20, 21]. A schematic feature of Sirt1 molecule is illustrated in Figure 2.

Sirt1 has been recognized as a chromatin silencing and stress response factor. The growing list of its substrates includes the tumor suppressor protein p53, members of the FoxO family (forkhead box factors), HES1 (hairy and enhancer of split 1), HEY2 (hairy/enhancer-of-split related with YRPW motif 2), PPARg (peroxisome proliferator-activated receptor gamma), CTIP2 (chicken ovalbumin upstream promoter transcription factor (COUPTF)-interacting protein 2), p300, PGC-1a (PPARg coactivator), and NF-κB (nuclear factor kappa B). Sirt1 is responsible for maintaining lysine deacetylation in histones and removing acetyl groups from many other histone and nonhistone proteins. It generates nicotinamide and acetyl groups from various substrates and turns them into 2′-O acetyl-ADP-ribose along with free nicotinamide products. These activities result in a range of biological effects such as cell survival, stress resistance, fat storage, insulin production, glucose homeostasis, lipid metabolism, and aging [22].

### 2.1. Activators and Inhibitors of Sirtuin 1

In addition to calorie restriction which has been established to improve Sirt1 enzymatic activity in human and mice [23], the most well-known molecular activators of Sirt1 are polyphenols such as resveratrol, quercetin, and epigallocatechin gallate (EGCG) which can increase Sirt1 activity up to the 8-fold, 2-fold, and 1.6-fold, respectively. Although no considerable side effect has been reported, poor absorption and rapid metabolism of these agents' affect their function and make the dose adjustment difficult to achieve [24]. Pharmacological Sirt1 activators which could be better handled in vivo include SRT1720, SRT2172, SRT2104, SRT2379, and metformin [25]. Besides, despite the lack of scientifically authenticated references about Sirt1-activating foods, the term “Sirtfoods” including green tea, red grape, olive, turmeric, parsley, broccoli, and some other products has been introduced as dietary source of Sirt1 activators.

The main Sirt1 inhibitors are sirtinol, splitomicin, AGK2, cambinol, suramin, tenovin, salermide, as well as lysine-based tripeptide analogues, nonpeptide containing *N*-thioacetyl lysine, thiobarbiturate, and indol derivative EX-527, most of them exerting their effect by occupying the catalytic core and preventing from proper substrate binding [15]. Nicotinamide (NAM), a form of vitamin B_3_, was first demonstrated as a Sirt1 inhibitor particularly in in vitro studies [5]; however, some in vivo studies showed that NAM could convert into NAD^+^, a cosubstrate of Sirt1, thus playing the role of Sirt1 activator [2]. For instance, it was found that NAM could promote hepatocytes regeneration in the animal model of liver transplant most likely by activating Sirt1 [26].

Because the feasibility of molecular manipulation is an important requirement for interventional studies and in view of the abovementioned list of available Sirt1 activators and inhibitors, this enzyme could be suggested as a suitable candidate for in vivo experiments.

### 2.2. Sirtuin 1 Effect on T Lymphocyte Subsets

T lymphocytes are considered as the main immune cells involved in allograft rejection and tolerance; therefore, newly introduced therapeutic agents' influence on T cell subsets should be examined [26, 27]. Chuprin et al. have demonstrated that Sirt1 is markedly expressed in mature Aire+ (autoimmune regulator) medullary thymic epithelial cells and is required for the expression of Aire-dependent TRA (tissue-restricted antigens)- encoding genes. Since the expression of these genes is necessary for eliminating self-reactive T cells, Sirt1 deficiency could increase the risk of autoimmunity. This finding proposes a considerable role for Sirt1 in T lymphocytes development [28]. It has also been shown that the Notch receptor in Treg cells is essential for survival of these cells in the calorie restriction condition, where the Notch1 intracellular domain remains close to the plasma membrane and has an antiapoptotic role (in contrast to its transcriptional activity when transferred to the nucleus). Sirt1 is suggested to stabilize the Notch1 intracellular domain proximal to the membrane and thereby promote Treg cells survival [29].

On the contrary, there are studies showing that Sirt1 deletion or inhibition could promote Treg cells activity via increased forkhead box P3 (Foxp3) transcription factor expression. Given the critical role of Treg cells in tolerance induction, Sirt1 overactivation may be detrimental to transplant. One of the studies supporting this idea was conducted by Beier et al. who demonstrated that Sirt1 deletion in mice improved Tregs suppressive activity in vitro and in vivo without affecting conventional T cells activation or proliferation. Mice with targeted deletions of Sirt1 in Foxp3+ Treg cells exhibited prolonged survival of mismatched cardiac allografts and more Treg cells infiltration in tissue. Sirt1 silencing by splitomicin and EX-527 in wild-type mice showed similar results [11]. Later, Levine et al. reported comparable findings by transplanting kidney from BALB/*c* mice into the Sirt1-deleted C57BL/6 recipients or mice treated with EX-527. Sirt1-deleted mice showed improved renal function and better graft survival compared to the WT recipients. The positive effect of Sirt1 inhibition with EX-527 was dose dependent as a very high concentration (10 mg/kg/day) did not exert any significant advantage [30]. Recently, Ye et al. evaluated the effect of sirtinol, a Sirt1 inhibitor, on heart allograft survival. They found that sirtinol-treated recipients had increased numbers of Foxp3 Treg cells. Moreover, CD4+ T cell isolated from sirtinol-treated recipients' blood showed reduced percentages of Th17 cells in addition to decreased expression of IL-17A and ROR*γ*t transcription factor; intracellular staining revealed lower amounts of IL-17 cytokine in comparison to DMSO-treated mice. However, IL-4 and IFN-*γ* expression did not alter significantly. Therefore, it was concluded that Sirt1 inhibition might affect Th17/Treg ratio in favor of Treg cells. Besides, a synergic effect was shown between tacrolimus and sirtinol; hence, they were suggested to apply together in order to improve efficacy [13]; a possible explanation of this finding is that Sirt1 inhibition increases Foxp3 acetylation which promotes the production and functions of Foxp3+ Treg cells, whereas acetylation of ROR*γ*t decreases its transcriptional activity and impairs the differentiation of Th17 cells [7]. It has also been demonstrated that Sirt1 inhibition decreased Foxp3 polyubiquitination and increased Foxp3 protein level. Moreover, cotransfection of Sirt1 with Foxp3 resulted in increased Foxp3 proteosomal degradation, while treatment with histone deacetylase inhibitors trichostatin A (TSA), nicotinamide(NAM), or MG132 increased the number of functional Treg cells [31, 32].

In contrast, Limagne et al. found that administration of Sirt1 activators like metformin could regulate IL-17A and ROR*γ*t expression via deacetylation of STAT3 transcription factor. STAT3 deacetylation restricts its binding ability to the Rorc promoter and diminishes Th17 cytokine secretion which consequently reduces vascular endothelial growth factor (VEGF) production and potentially tumor suppression [33]. According to these controversial findings, it could be hypothesized that endogenous activation of Sirt1 promotes Th17 function by deacetylation of Rorc, whereas pharmacological activation of Sirt1 may inhibit Th17 differentiation by STAT3 deacetylation; certainly, more studies are required to define appropriate doses of Sirt1 activators to inhibit Th17 cells activation in allograft.

In addition to Treg and Th17 cells, other T lymphocyte subtypes association with Sirt1 activation has been studied; for instance, Zou et al. showed that resveratrol could inhibit TCD4+ lymphocytes activity and reduce IL-2, IFN-*γ*, IL-4, and IL-5 cytokine production in vitro and in vivo. Moreover, IgM secretion was shown to be reduced and c-Jun translocation from plasma to the nucleus was significantly arrested following Sirt1 activation [12]. A previous study had demonstrated Sirt1 involvement in tolerance induction and clonal T cells energy as it was observed that Sirt1 deletion resulted in T CD4+ cells proliferation and excessive IL-2 expression, whereas Sirt1 activation could suppress T cells function. In addition, Sirt1-deficient mice had impaired T cell tolerance and were susceptible to severe experimental allergic encephalomyelitis (EAE); thus, Sirt1 activators like resveratrol were suggested as supplementary therapeutic agents for treating autoimmunity [34]. In fact, the role of Sirt1 in regulating differentiation and function of various T cell subsets has not yet been fully understood; therefore, further studies are required to clarify how T cells are influenced by Sirt1 in various stages of development and to define optimal levels of Sirt1 activity for modulating T cells function in favor of transplant (Table 1).

### 2.3. Sirtuin 1 and Protection against Ischemia Reperfusion Injury

Ischemia reperfusion injury (IRI) is considered as one of the main obstacles in achieving successful transplantation; therefore, treating the allograft with substances which could improve tissue maintenance and hypoxia resistance might be helpful in promoting clinical outcome; Sirt1 may have a role in this respect. It has been shown that expression of Sirt1 mRNA and protein was reduced in ischemia reperfusion (I/R) [8].

Hsu et al. compared cardiac specific Sirt1-knockout mice with cardiac-specific Sirt1-overexpressing ones and observed a significant increase in myocardial infarction size in cardiac-Sirt1−/− mice comparing to the overexpressing group; upregulated expression of prosurvival molecules, MnSOD, thioredoxin1, and Bcl-xL, along with downregulation of proapoptotic molecules Bax and cleaved caspase-3 was reported in Sirt1+ animals. Besides, they studied oxidative stress in ischemic tissue using 8-OHdG staining and found a negative association between Sirt1 expression and oxidative stress [35]. Similarly, Nadtochiy et al. comparing SIRT1-deficient (SIRT1^+/−^) and SIRT1-overexpressing (SIRT1^+++^) mice in heart ischemic preconditioning (IPC) showed that SIRT1^+/−^hearts could not be preconditioned properly, whereas SIRT1^+++^ hearts were endogenously protected against ischemia reperfusion injury and exhibited decreased cytosolic acetylation which was reversible by SIRT1 inhibitor splitomicin [36]. Calorie restriction as a Sirt1 stimulator has also been demonstrated to induce heart ischemia tolerance [37]. These and many other studies on heart ischemia models suggest a considerable protective role for Sirt1 [9].

Later, Liu et al. infected aged mesenchymal stem cells (MSCs) with lentivirus particles carrying Sirt1 plasmids. They assigned three groups of rats: 20 received intramyocardial injection of cell culture medium (DMEM), 20 got MSCs carrying empty vectors, and 20 were treated by MSC-Sirt1. After 1 and 3 days of ischemia induction, the group receiving MSCs-Sirt1 showed significantly improved blood vessel density in the infarction region and exhibited better cardiac function comparing to the other groups. In vitro study demonstrated increased expression of proangiogenic factors angiopoietin 1 (Ang1) and basic fibroblast growth factor (bFGF), together with reduced mRNA level of antiangiogenic factor thrombospondin-1 (TBS1) in MSCs-Sirt1; moreover, the Bcl-2/Bax ratio was elevated. These results showed that Sirt1 gene transfection could restore angiogenesis capacity of aged mesenchymal stem cells and improved their efficacy in protection against ischemic injury [38]. To study the Sirt1 role in resuscitation following hemorrhagic injury, Ayub et al. administrated resveratrol and its synthetic mimic SRT1720 during the resuscitation of rats. Sirt1 activation using these agents prolonged the survival of rats in the absence of resuscitation fluid and restored left ventricular function significantly demonstrated by tissue expression of mitochondria-related transcription factors Ppar-*α*, Tfam, and peroxisome proliferator-activated receptor gamma coactivator-1 (PGC-1*α*) [39].

Nakamura and colleagues demonstrated that heme oxygenase-1 (HO-1) could play a cytoprotective role against IRI by triggering a macrophage specific HO-1-Sirt1-p53 signaling pathway. They showed that lower HO-1 expression in human postreperfusion liver transplant biopsies is associated with increased liver enzymes and decreased recipient survival; moreover, Sirt1 and p53 expression reduced, while cleaved caspase-3 increased significantly in low HO-1 expressing patients. They also studied the effect of myeloid-specific HO-1 deletion on the murine model of hepatic ischemia and observed considerable hepatocyte apoptosis and deteriorated tissue inflammation due to the lack of Sirt1. On the contrary, myeloid-specific HO-1 overexpression ameliorated inflammation and reduced ischemia reperfusion injury. Furthermore, I/R-stressed livers of HO-1 KO mice when conditioned with resveratrol restored p53 signaling and showed improved outcomes [40]. Rickenbacher et al. investigated the association between Sirt1 expression and IRI in the mice liver ischemia model. The mice which underwent one-day fasting showed significantly better liver function and less proinflammatory cytokine including TNF-*α*, IL-1b, IL-6, and CCL2, following IRI in comparison to the control (sham food) group. In addition, expression of monocyte differentiation marker CD14 and the number of Mpo-positive neutrophils were reduced markedly in fasting liver tissue. Serum high mobility group box 1 (HMGB1) levels also showed significant decrease. To assess Sirt1 contribution to this finding, the mice were treated with Sirt1 antagonist EX-527. EX-527 neutralized protective effect of fasting and restored HMGB1 levels up to the normal range. Additionally, in vitro study by incubation of the macrophage-like cell line Raw264-7 in fasting-mimicking condition did not affect their viability; however, TNF-*α* secretion and intracellular ROS production decreased in both unstimulated and LPS-challenged cells. Therefore, it was concluded that Sirt1 expression protects against IRI by suppressing HMGB1 and other inflammatory mediators [41].

In kidney transplantation, Giovannini et al. demonstrated that resveratrol administration could reduce renal damage in ischemic rats according to the histological examination and serum creatinine evaluation; resveratrol was also shown to inhibit lipid peroxidation induced by ischemia/reperfusion in the renal cortex and medulla [42]. In agreement with this study, Chander and Chopra could attenuate renal dysfunction and morphological alterations after ischemia reperfusion by pretreatment of animals with resveratrol; moreover, Sirt1 activation was found to restore depleted renal antioxidant enzymes [43]. Fan et al. observed that IRI could induce considerable renal injury in adult mice (4-month-old) but only mild damage in younger ones (2-month-old), whereas administration of Sirt1 activator, SRT1720, in adult mice improved renal tissue maintenance considerably. In addition, they showed that genetic ablation of one Sirt1 allele (SIRT1(±)) could deteriorate IRI comparing to the wild type (SIRT1(+/+)) [44]; in a similar study, treating the mice with SRT1720 at the time of reperfusion resulted in better renal histological architecture and reduced apoptosis and less creatinine levels compared to the vehicle carriers (20% dimethyl sulfoxide in saline). Renal adenosine triphosphate (ATP) levels and mitochondrial mass which reduce due to I/R restored considerably in response to Sirt1 activation. Furthermore, malondialdehyde, nitrotyrosine, iNOS and TNF-*α* expression, macrophage infiltration, IκB-*α* degradation, and NF-ΚB phosphorylation decreased in SRT1720-receiving mice [45]; nonetheless, later Bienholz and colleagues studying some groups of renal IRI animal models—with and without resveratrol application in low and high doses—found no significant difference between resveratrol-receivers and other groups in histopathological features and serum chemical determinants. They attributed this finding to the blood pressure instability following I/R in the resveratrol group which required more bolus injection and induced TNF-*α* expression [46].

In intestinal ischemia reperfusion injury, treating the mice with SRT1720 at reperfusion time improved tissue microscopic architecture reduced apoptosis and decreased intestinal TNF-*α* and iNOS mRNA levels to the baseline. In addition, systemic inflammation, as determined by serum IL-6 level, was reduced [47]. These and other findings indicate Sirt1 probable potential in stabilizing allograft tissue during ischemia reperfusion injury [8].

### 2.4. Anti-Inflammatory Effects of Sirtuin 1

There are some experiments demonstrating Sirt1 implication in regulating immune responses in vitro and in vivo [26]. Sirt1 expression has been shown to attenuate inflammatory responses in chronic obstructive pulmonary disease (COPD), cardiac hypertrophy, and inflammation-associated liver diseases. Sirt1 overexpression ameliorates the condition, while its gene silencing results in inflammatory diseases exacerbation [10]; moreover, Sirt1 expression is reduced in the inflammatory environment [6]. The underlying mechanism for Sirt1 anti-inflammatory effect is supposed to be NF-κB and AP-1 molecules regulation, as it has been demonstrated that Sirt1 affects transcriptional activity of NF-κB by interfering with its RelA/*p*65 subunit and deacetylating lysine 310 at this site. Since NF-kB is one of the main transcription factors in cytokine production and immune responses, its inhibition would result in immunomodulation [48]. In addition, Sirt1 directly interacts with c-Jun and decreases c-Fos/c-Jun acetylation and, therefore, inhibits the transcriptional activity of AP-1 and subsequently suppresses MMP9 and COX-2 expression [49]. Other inflammation-related transcriptional factors which are deacetylated and inactivated by Sirt1 are peroxisome proliferator-activated receptor (PPAR), PGC-1, and FoxO [10].

To define the anti-inflammatory properties of Sirt1, Yue et al. generated heme oxygenase-1 (HO-1)-overexpressing bone marrow-derived macrophages using adenoviral vectors (Ad-HO-1) and infused them into the liver grafts via portal vein prior to reperfusion. Compared to the control Ad-*β*-gal-macrophage receivers, HO-1-overexpressing mice exhibited less tissue injury, lower ALT level, lesser neutrophil count, and increased TGF-*β*/IL-10 expression. Moreover, macrophage differentiation was shifted from M1 (Nos2+) to M2 (Mrc-1/Arg-1+) phenotype. Of note, Sirt1 expression was markedly elevated in HO-1-expressing group, whereas Sirt1 inhibition restored reperfusion-induced injury despite Ad-HO-1 macrophages transfer [50]. Similarly, Nakamura et al. evaluated liver function as well as pro- and anti-inflammatory cytokine levels in the liver IRI model of wild type and HO-1 knockout (HO-1 KO) mice. Sirt1 expression was significantly reduced in the HO-1 KO mice liver, while ALT and AST enzymes amount showed elevation. Inflammatory mediators mRNA level including IL-6, IL-1*β*, IFN-*γ*, TNF-*α*, MCP-1, IP-10, MIP-2, and CXCL-1 increased, whereas anti-inflammatory cytokines IL-10 and TGF-B mRNA decreased compared with the wild type [51]. Further studies showed that HO-1 regulates macrophage activation via Sirt1-p53 signaling network, and manipulating this pathway was suggested as a therapeutic modality to protect liver allograft from IRI and subsequent inflammatory alloresponses [52].

Yoshizaki and colleagues demonstrated that Sirt1 represses proinflammatory gene expression including TNF-*α*, IL-6, MCP-1, JNK, VCAM, MMP9, CRP, IL-1B, and COX2 because siRNA inhibition of Sirt1 gene in 3T3-L1 adipocytes resulted in significant increase of mentioned genes mRNA level. Moreover, following adipocyte treatment by Sirt1 activators SRT2530 and SRT1720a, significant anti-inflammatory effect was observed which protected adipocytes against insulin resistance. This finding was attributed to NF-κB inhibition [53]. They also reported that Sirt1 knockdown in mouse macrophage RAW264.7 cell line and intraperitoneal macrophages resulted in marked activation of the JNK and IKK inflammatory pathways and an increased gene expression of TNF-*α*, IL-1b, MMP9, MCP-1, KC, and IL-6. To confirm these findings, they showed that Sirt1 activators, SRT1720 and SRT2379, inhibited LPS-stimulated inflammatory pathways and suppressed TNF-*α* secretion from macrophages [54]. In liver transplantation, resveratrol-treated mice displayed elevated Sirt1 expression, less leukocyte infiltration, lower proinflammatory mediators (COX-2, iNOS, interleukin-1*β*, MCP1, TNF-*α*, and CXCL10) secretion and better graft function. In vitro study of bone marrow-derived macrophages (BMDMs) showed that Sirt1 silencing (using siRNA in cell culture) enhanced inflammatory cytokine expression, whereas adding resveratrol inhibited macrophage activation, decreased the proinflammatory response, and suppressed IFN-*γ* production in coculture with liver infiltrating and spleen lymphocyte cells. In addition, human liver transplant recipients with higher tissue expression levels of Sirt1 had improved survival rates, lower levels of COX-2 and T-bet transcripts, as well as reduced expression of proinflammatory cytokines (IL1*β*, MCP1, TNF-*α*, CXCL10, and IFN-*γ*) [40]. Taken together, Sirt1 and its activators may be beneficial for preventing from alloimmune responses in transplantation by suppressing proinflammatory mediators expression.

### 2.5. Sirtuin 1 in Stem Cell and Solid Organ Transplantation

#### 2.5.1. Sirtuin 1 and Stem Cell Transplantation

In 2012, Leko et al. compared outcomes of bone marrow transplantation from Sirt1−/− to WT mice with a usual WT to WT BM transplant. Apart from anemia and leukocytosis among older animals, Sirt1 deletion affected neither frequency of hematopoietic stem cell (HSC) populations in BM nor production of mature blood cells, so they concluded that Sirt1 is not essential for stem cells maintenance and maturation [55]. However, Singh et al. demonstrated that loss of Sirt1 in mice resulted in HSCs aberrant expansion; in addition, Sirt1-deficient hematopoietic progenitors showed increased DNA damage, p53 overactivation, and genomic instability; therefore, they concluded that Sirt1 is necessary for stem cells and progenitor cells maintenance and regulation in bone marrow [56].

On the contrary, stem cells appear to be a source of Sirt1; for instance, it has been shown that senescence-accelerated mice prone 10 (SAMP10) model ameliorated by allogenic intrabone marrow transplantation as Sirt1, autoimmune regulator (Aire), and keratinocyte growth factor (KGF) insufficiency in thymus epithelial cells was resolved [57]; later, intrabone marrow plus thymus transplantation was performed for mice model of nonalcoholic fatty liver disease which resulted in decreased levels of IL-6, MCP-1, leptin, and LDL in plasma, increased Sirt1 and HO-1 expression, and decelerated disease progression [58].

Recently, stem cells transplantation (infusion) has emerged as a novel treatment for tissue repair following heart and brain strokes. Liu et al. transplanted SRT1720-treated aged human mesenchymal stem cells (MSCs) into rats' myocardial infarction model and found enhanced left ventricular ejection fraction, improved angiogenesis, and reduced fibrosis of heart tissue compared to DMSO-treated MSCs infusion. Upregulated expression of Fas apoptosis inhibitory molecule (FAIM) in SRT1720-treated MSCs was presumed as the possible mechanism for protective effect of Sirt1 in the ischemic model [59]. Similarly, Ozawa et al. performed skeletal stem cell transplantation from young, adult, and Sirt1-KO (using siRNAs for SIRT1) mini-pigs into the cardiac failure model. They showed better cardiac function along with less myocardial fibrosis in stem cell transplantation from juvenile mice compared to adults. Expression of Sirt1, hypoxia-induced factor-1a, hepatocyte growth factor, and stromal cell-derived factor-1 was significantly upregulated in young stem-cell receivers' heart tissue. Transplantation from Sirt1-KO stem cells caused less functional improvement compared to the other groups. Moreover, in vitro study showed less proliferation potential and more cytokine secretion from Sirt1-KO cells compared to the wild type [60]. Taken together, either intrinsic Sirt1 expression by stem cells or Sirt1 overactivation induction in stem cells could be considered as a therapeutic option for treating certain diseases as well as improving stem cell transplantation (Table 2).

#### 2.5.2. Sirt1 and Liver Transplantation

Pantazi et al. comparing a liver preservation solution with its trimetazidine- (TMZ-) enriched form studied Sirt1 effect in tissue protection. Given that TMZ promotes nicotinamide phosphoribosyltransferase (NAMPT) expression and improves NAD cycling which increases the NAD+ level, it is supposed to induce Sirt1 activity. Gene expression assay revealed enhanced Sirt1 expression in tissue preserved in TMZ-enriched graft which resulted in less p53 and FoxO expression and lower plasma levels of alanine aminotransferase (ALT) and hepatic glutamate dehydrogenase (GDH). Although these effects were significantly correlated to Sirt1 activation, it was shown that heat shock protein 70 (HSP70) and AMP-activated protein kinase (AMPK) increased in presence of TMZ. Since HSP70 has a protective role in IRI and AMPK regulates mammalian target of rapamycin (mTOR) which is involved in T cells activation, improved transplant outcome could be attributed to the combination of these three pathways effect [61]. The same group administrated losartan which has been shown to induce Sirt1 gene upregulation, to the donor and recipient rats 24 h and 1 h before liver transplantation. 24 hours after reperfusion, Sirt1 expression and activity along with NAD+/NADH and NAMPT levels increased, while FoxO1 protein expression decreased significantly in losartan-receivers compared to the control group. These animals also displayed lower ALT and aspartate aminotransferase (AST) serum levels and less mitochondrial injury. Moreover, caspase 12 and cleaved caspase 3 amounts were reduced significantly by losartan administration, the finding which represents less apoptosis in liver allograft tissue [62]. Despite these promising findings, obviously further studies are required to provide sufficient evidence to suggest Sirt1 as a potent tissue protective substance in liver transplantation (Table 2).

### 2.6. Sirtuin 1 and Renal Tissue Maintenance

Sirt1 is constitutively expressed in medullary as well as cortical proximal tubular cells and has been shown to be involved in renal tissue maintenance; this enzyme is supposed to protect renal tissue against ischemic and toxic injuries [63]. To support this idea, He et al. demonstrated that the unilateral ureteral obstruction (UUO) model of kidney injury results in more renal apoptosis and fibrosis in Sirt1+/–mice than in WT controls; moreover, SRT2183, a Sirt1 activator, significantly reduced apoptosis and fibrosis in WT mice. They also observed a significant correlation between Sirt1 activation and oxidative stress-induced cyclo oxygenase-2 (COX-2) expression in both in vitro model of cultured mouse renal medullary interstitial cells and in vivo. Since COX-2 expression promotes blood circulation, they attributed protective effects of Sirt1 to some extent to COX-2 upregulation [64]. To investigate Sirt1 protective role against acute kidney injury (AKI), Hasegawa and colleagues created transgenic mice with kidney-specific overexpression of Sirt1 that were treated with cisplatin for three days. Cisplatin-induced AKI reduced the number and activity of peroxisomes and mitochondria, led to increased production of local reactive oxygen species (ROS) production, and caused renal tubular apoptosis in normal mice. However, transgenic mice did not have fewer peroxisomes and did have better mitochondrial and peroxisomal function. Similar effects were observed by calorie restriction which is considered to induce Sirt1 expression. Nonetheless, overexpression of Sirt1 failed to protect against IRI since neither renal function (assessed by BUN and creatinine) nor histological findings were improved in Sirt1-transgenic mice [65].

### 2.7. Sirtuin 1 Dual Role in Cancer

Sirt1 has been shown to have both tumor suppressor and tumor promoter properties depending on its deacetylation target [14]. For instance, deacetylation of p53 (in Lys382 residue) and p73 as tumor suppressor factors would be predicted to result in a higher risk of cancer [66]. In contrast, sirtuin 1 is mandatory for maintaining genomic stability, as chromosomal aberrations are significantly increased in Sirt1-deficient cells increasing their sensitivity to tumorigenic mutations [67]. Sirt1 overexpression in prostate cancer, acute myeloid leukemia, primary colon cancer, skin squamous cell carcinoma, basal cell carcinoma, Bowen's disease, and actinic keratosis has been reported [22]. According to these observations, the notion of general or local Sirt1 overexpression should be considered carefully.

## 3. Conclusion

Sirt1 is the most prominent member of the sirtuin family of histone deacetylases and is known to be involved in the regulation of several genes and in the function of many molecules [16]. In addition to the implication of Sirt1 in cell survival, aging, and metabolism, recent studies have demonstrated an anti-inflammatory function and protective effects against ischemia reperfusion injury [8, 10]. Moreover, it has been shown that Sirt1 plays a role in tissue maintenance and repair particularly in the liver and kidney [63]. Because of these observations, Sirt1 could be suggested as a promoting factor in transplantation tolerance; thus, its organic and synthetic activators could be considered as supplementary agents to protect and preserve donated tissue. Despite the elaborated list of available Sirt1 activators [15], there are remaining concerns about Sirt1 overexpression and overactivation in vivo. The first concern is the implication of Sirt1 in inactivating tumor suppressor molecules such as p53 [14]. In spite of potentially positive consequences of p53 suppression in tissue maintenance during ischemia reperfusion injury, excessive p53 suppression might increase the risk of tumorigenic alterations leading to development of malignancies. The second concern is the unclear role of Sirt1 in T lymphocyte subsets differentiation and function. There are studies suggesting that Sirt1 impairs the Treg/Th17 ratio in favor of Th17 which could negatively affect the outcome of transplantation due to the implication of Th17 cells in chronic inflammatory responses [13]. Nonetheless, there are findings proposing Sirt1 as an essential factor for the development of central (thymic) tolerance [12] and for Treg survival in calorie restriction condition [29]. Moreover, Sirt1 activation has been found to result in overall suppression of TCD4+ lymphocytes which could be beneficial for protecting the allograft from cellular rejection [12, 34]. In order to clarify the impact of Sirt1 on T cells and to define the pros and cons of Sirt1 overactivation on T cell subsets' balance, it is suggested to perform in vitro experiments treating different T cell subsets by various Sirt1 activators and inhibitors in different doses in pure and mixed culture conditions. The next step could be introducing the treated cells into animal models to evaluate the sustainability and duration of the in vitro effects in vivo.

Despite these two drawbacks, there are many studies demonstrating the positive effects of Sirt1 activation on graft survival and transplant promotion. These effects could be classified in three branches of protection from ischemia reperfusion injury, anti-inflammatory properties, and tissue repair potential. The protective role of Sirt1 against ischemia reperfusion injury has been shown by several studies and can be considered as the most described aspect of its participation in transplant improvement [8]; hence, it might be an appropriate option for conducting advanced investigations and clinical trials. According to the previous experiments, Sirt1 could be overexpressed, deleted, overactivated, or inhibited either in in vitro or in vivo systems. Because gene deletion/insertion or suppression/overexpression studies are not easily feasible for human recipients, the most practical manipulation tool might be the administration of natural or pharmacological activators (or inhibitors) of Sirt1 such as resveratrol (or sirtinol) to the recipients which obviously requires meticulous safety authorization and dose adjustment beforehand. Determining the exact amount of Sirt1 activators in so-called “sirtfoods” and administrating calculated doses of them in the daily nutritional regimen might be the most simple and safe way to start with. Subsequent evaluation of immune elements alteration in case of clinically significant findings would be necessary.

Most evidence about anti-inflammatory effects of Sirt1 has been collected from liver transplantation studies [40]. In vitro experiments with mice macrophage RAW264.7 and 3T3-L1 adipocytes cell line has also shown regulatory effect of Sirt1 on proinflammatory genes expression [53, 54]; this effect has been explained by inhibition of the principal inflammatory transcription factors NF-κB and AP-1 by Sirt1 [48]. Given that the control of inflammatory responses is an essential requirement for graft survival, these and similar findings could propose Sirt1 as a candidate for designing novel supplementary immunosuppressive agents; in particular, liver transplant recipients may profit from anti-inflammatory and tissue preserving properties of Sirt1 if its natural or pharmacological activators are included in their immunosuppressive drugs.

Despite positive results obtained from the injection of Sirt1+ mesenchymal stem cells for tissue regeneration in animal models [59, 60] there are almost no solid findings about its efficacy in human patients. The potential of Sirt1 in tissue repair and maintenance suggest it as a candidate for manipulating mesenchymal stem cells and transferring them into the damaged tissue particularly in heart stroke. Moreover, Sirt1 activators could be considered as supplementary agents for tissue repair after the injurious procedure of solid organ transplantation.

As mentioned before, generally, there are two forms of increasing Sirt1 activity: overexpression and overactivation, the latter being more feasible for clinical experiments; however, Sirt1 overexpression might be proposed as an option for improving the viability and accommodation of graft tissue in tissue engineering and replacing damaged tissue by ex vivo expanded or developed tissues. At the moment, there are no data about such an exploit in designing artificial organs, but there are evidence of using Sirt1 as a differentiation factor for generating osteocyte [28] and chondrocytes [7] from mesenchymal stem cells.

As Sirt1 is involved in a wide range of biological and pathological events, any experiment examining a unique aspect of its effects on allograft may reveal a particular positive or negative result, which may explain the discrepancy between outcomes of some studies. Therefore, it is necessary to explore its various (tissue preserving, anti-inflammatory, and malignancy inducing) effects on a single system to reach a more impartial and practical insight into the role of Sirt1 in transplantation. Thereafter, we should try to provide a comprehensive explanation for each finding with the aim of augmenting the favorable effects and resolving the adverse ones.

In summary, Sirt1 might be considered as an auxiliary factor for the protection of allograft tissue against ischemia reperfusion injury and inflammation as well as for tissue maintenance posttransplant; however, there is a risk that Sirt1 overexpression could predispose the tissue to malignant alterations. In addition, despite Sirt1 involvement in tolerance induction and T cell suppression, impaired Treg/Th17 balance may be an adverse outcome of Sirt1 overexpression. Definitely, further studies are required to explore pros and cons of Sirt1 overactivation in transplanted tissue and their mechanisms.

## Figures and Tables

**Table 1:** Sirtuin 1 effects on T cell subsets.

Treg	Sirt1 stabilizes Notch1 intracellular domain proximal to the membrane ⟶ promotes Treg cells survival and function (29) Sirt1 deletion or silencing by splitomicin and EX-527 ⟶ prolonged cardiac allograft survival in a Treg-dependent manner (11) Sirt1 CD4 conditional deletion of Sirt1 (Sirt^fl/fl^CD4^cr^) or EX-527 administration ⟶ improved renal allograft outcome (30) Sirt1 inhibition by sirtinol in heart TX⟶ increased proportion of Treg cells (13) Sirt1 inhibition ⟶ decreased Foxp3 polyubiquitination and degradation, increased Foxp3 protein level (31)

Th17	Sirt1 inhibition by sirtinol in heart TX ⟶ reduced percentage of Th17 cells; decreased expression of IL-17A and ROR*γ*t transcription factor (13) Sirt1 activation by metformin in tumor⟶ reduced IL-17A secretion and ROR*γ*t expression (33)

T CD4+ lymphocytes	Sirt1 deficiency in thymus ⟶ impaired elimination of autoreactive T cells (28) Sirt1 activation by resveratrol ⟶ inhibited T cells activity, reduced cytokines IL-2, IFN-*γ*, IL-4, and IL-5 production and inhibited c-Jun translocation to the nucleus(12) Sirt1 deletion ⟶ T CD4+ cells proliferation (34) Sirt1 activation by resveratrol ⟶ inhibited T cells activation and improved tolerance (34)

Sirt1: sirtuin 1; TX: transplantation; Th: T helper; IL: interleukin; IFN-*γ*: interferon-*γ*

**Table 2:** Sirtuin 1 effects on stem cells and liver transplantation.

Stem cell transplantation	Sirt1−/− bone marrow TX ⟶ normal output (55) Sirt1−/− bone marrow TX ⟶ stem cells aberrant expansion, increased DNA damage, p53 overactivation, and genomic instability (56) Intrabone marrow-bone marrow TX ⟶ amelioration of SAMP10 and mice nonalcoholic fatty liver disease (57, 58) SRT-1720-pretreated-mesenchymal stem cells infusion to myocardial infarction model ⟶ left ventricular ejection fraction↑, angiogenesis↑, fibrosis↓(59) Sirt1-KO skeletal stem cell TX infusion to myocardial infarction model ⟶ functional improvement↓, proliferation rate↓, cytokine secretion↑(60)

Liver transplantation	Liver preservation in TMZ ⟶ Sirt1 activation ⟶ p53 and FoxO expression↓, ALT and GDH↓, improved graft survival (61) Losartan administration before liver TX ⟶ Sirt1↑, FoxO1↓, ALT and AST↓, caspase 12 and cleaved caspase 3↓(62)

Sirt1: sirtuin 1; TX: transplantation; SAMP10: senescence-accelerated mice prone 10; TMZ: trimetazidine; ALT: alanine aminotransferase; AST: aspartate aminotransferase; GDH: glutamate dehydrogenase.

## Abbreviations

Sirt1: Sirtuin 1
HSCT: Hematopoietic stem cell transplantation
MSCs: Mesenchymal stem cells
Th: T helper
Treg: T regulatory
NAD: Nicotinamide adenine dinucleotide
IRI: Ischemia reperfusion injury
HO-1: Heme oxygenase-1.

## Supplementary Materials

Supplementary Materials Graphical Abstract.
