# Supplementary figures and images for: Sirtuin 1: A Dilemma in Transplantation
Source: J Transplant. 2020 Apr 25;2020:9012980. doi: 10.1155/2020/9012980 (PMC7196964; doi:10.1155/2020/9012980)

## Graphical abstract

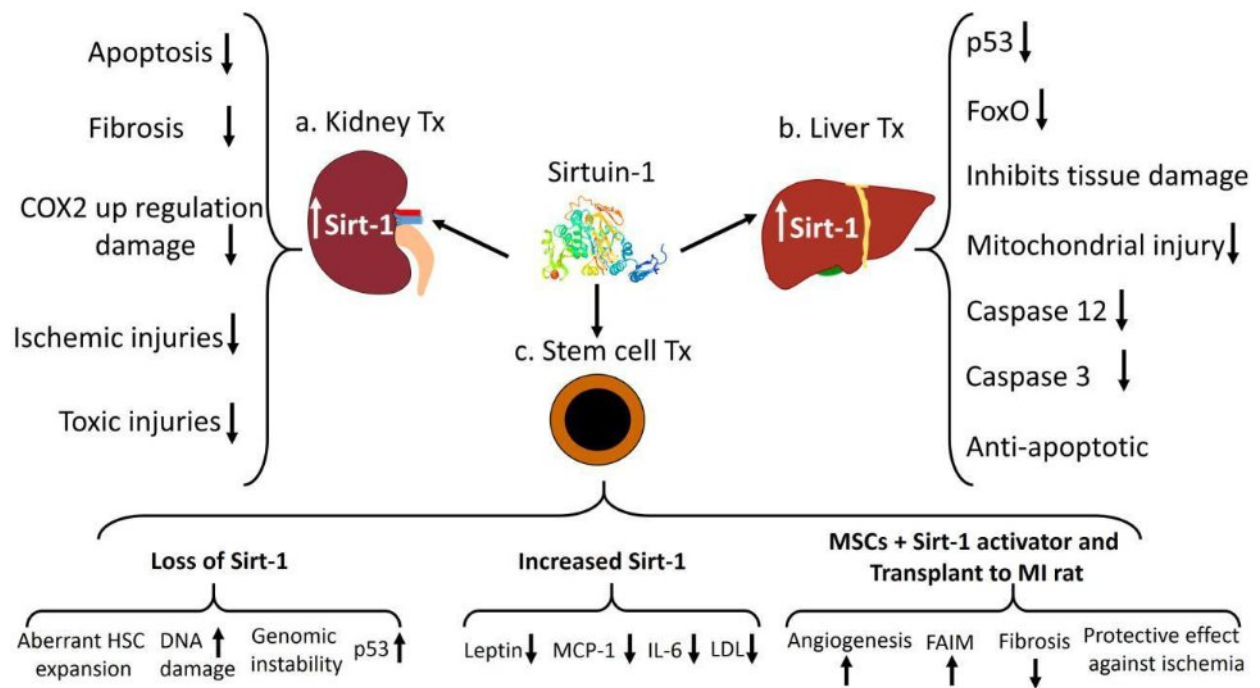

Supplement: Supplementary Materials — Graphical Abstract. [file 9012980.f1.pdf]
